# Skeletal Muscle Metastases in a Patient With Neuroendocrine Tumor

**DOI:** 10.4021/wjon609w

**Published:** 2013-05-06

**Authors:** Wilhelmina delos Santos-Cabalona, Olga N. Kozyreva, Ashley Davidoff, Gail Wolfe, Alan Hackford

**Affiliations:** a St. Elizabeth Medical Center, 736 Cambridge St, Boston, MA 02135, USA

**Keywords:** Carcinoid tumor, Neuroendocrine tumor, Muscle metastasis

## Abstract

Carcinoid tumors are rare but diverse group of malignancies that arise from neuroendocrine cells. Skeletal muscle metastasis is exceedingly rare and is associated with a poor prognosis. We report a case of carcinoid tumor of the ileocecal with skeletal muscle metastasis. We also review available case reports of carcinoid tumors metastasizing to the muscle.

## Introduction

Carcinoid tumors are neuroendocrine tumors derived from enterochromaffin cells and often spread via lymphatics or a hematogenous route to the lymph nodes, liver and bones. Metastases to skeletal muscle are exceedingly rare with only several cases reported. We present a case of primary ileocecal carcinoid metastasizing to the vastus intermedius muscle detected by somatostatin receptor scintigraphy and review the literature of this specific site of metastasis.

## Case Report

**Figure 1:**
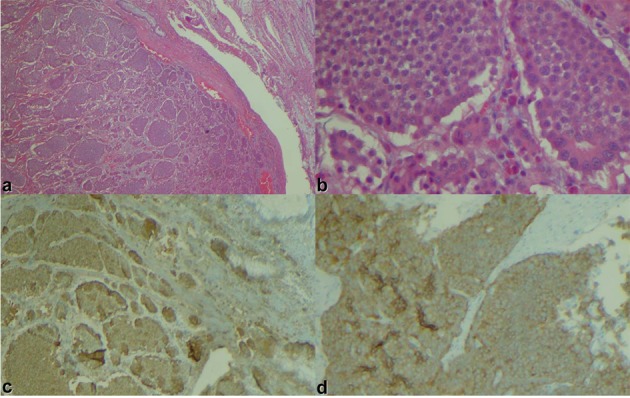
Small intestinal tumor in (a) low power view, (b) high power view, (c) chromogranin stain, (d) synaptophysin stain.

**Figure 2:**
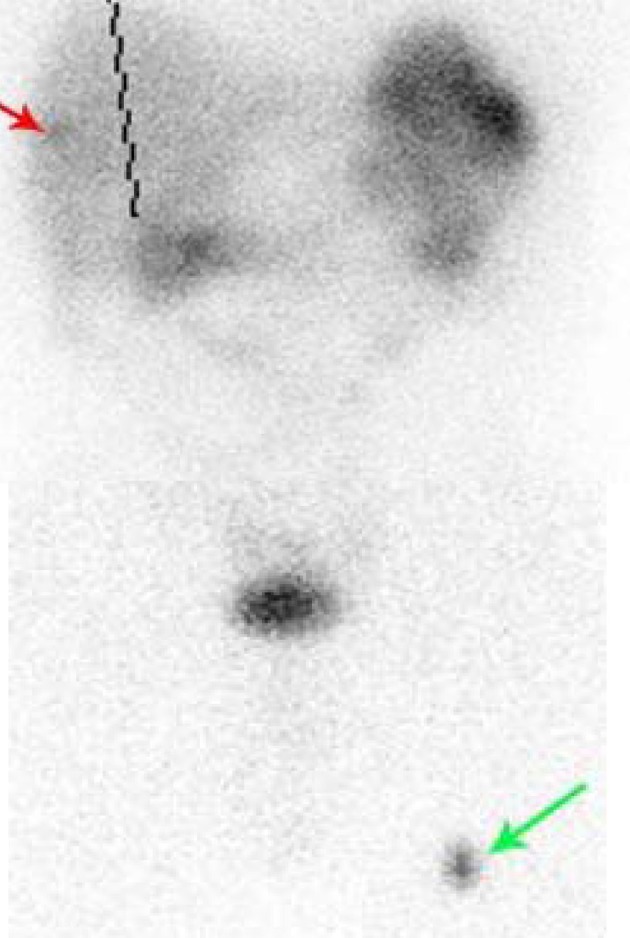
Octreoscan showing metastasis to liver (red arrow) and thigh (green arrow).

**Figure 3:**
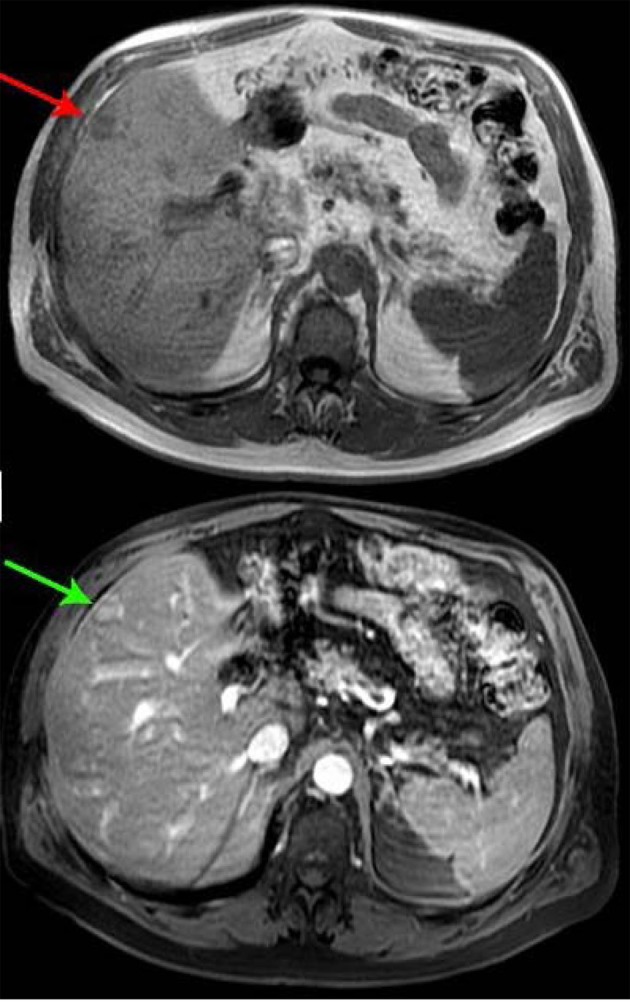
Abdominal MRI.

**Figure 4:**
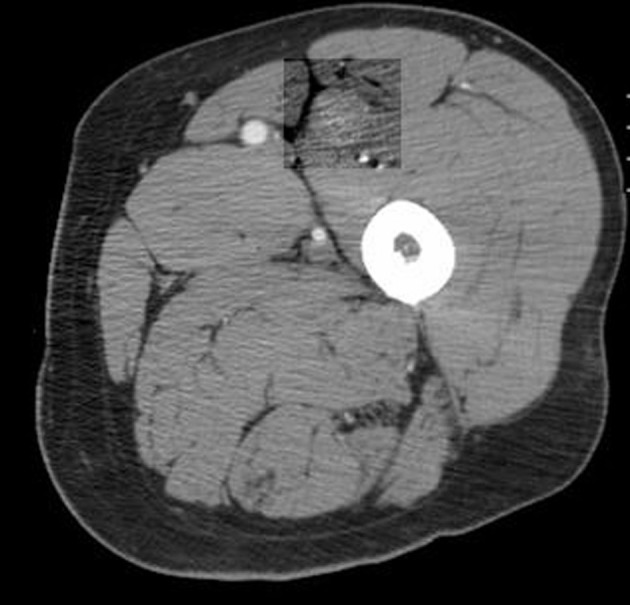
CT scan of the left thigh.

**Figure 5:**
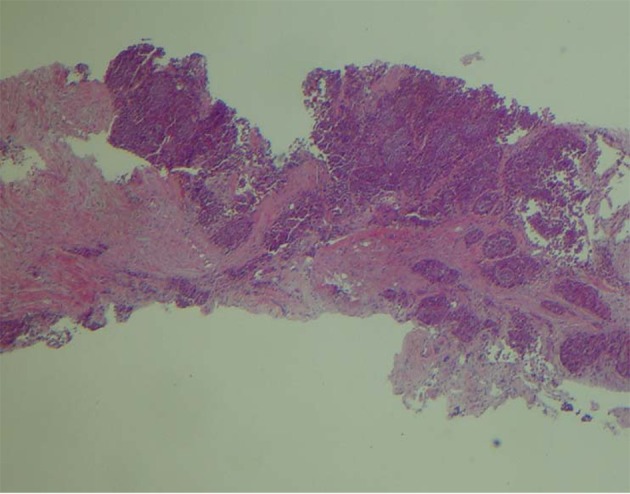
Low power view of the biopsy of left thigh.

A 66-year-old male presented with small bowel obstruction due to a cecal mass requiring exploratory laparotomy. Pathology revealed a well-differentiated neuroendocrine tumor with regional nodal metastases (Figure 1 shows small intestinal tumor in low power view (a), high power view (b), chromogranin stain (c), synaptophysin stain (d)). Eight months after surgery the patient presented with persistent diarrhea. Indium 111 octreotide scintigraphy (OctreoScan®) revealed increased activity in the left leg and right lobe of the liver (Figure 2 with green arrow shows metastasis to the liver, red arrow showing metastasis to left thigh). An abdominal MRI demonstrated a 2 cm subcapsular lesion at the right hepatic lobe (Fig. 3) and CT scan demonstrated an enhancing soft tissue mass in the left vastus intermedius muscle measuring 30 × 17 × 16 mm (Fig. 4). Biopsy of the left thigh lesion showed metastatic well-differentiated neuroendocrine tumor (Fig. 5). Subsequent CT scan demonstrated increase in the size of the liver and muscular lesions and Sandostatin® LAR was initiated.

## Discussion

The skeletal muscles comprise approximately 43-50% of the body mass, are well vascularized, and receive a large proportion of the cardiac output. Vascular embolization is a common modality for tumor metastasis. Despite this, hematogenous metastases to the skeletal musculature are relatively rare [1, 2]. Several factors have been implicated in the rarity of this finding including high blood turbulence, muscle motion affecting tumor implantation, high levels of lactic acid, and the presence of proteases and other inhibitors that creates a hostile environment for cancer cells to home in [2-4]. Metastases to skeletal muscles is reported in the setting of advanced malignancy, with lung cancer, renal cancer, melanoma and gastrointestinal tract adenocarcinoma as the most frequent primary sites of cancer [1, 2]. The prognosis associated with skeletal muscle metastases is poor, consistent with the fact that it represents a systemic disease. In the two large series the mean survival from diagnosis of muscle metastasis reported ranges from 5.9 to 8 months [5, 6].

Neuroendocrine (NETs) tumors are rare but diverse group of malignancies that arise from neuroendocrine cells, more commonly in the gastrointestinal tract. According to a recent analysis of the Surveillance, Epidemiology and End Results (SEER) database, the estimated annual incidence of carcinoid tumors is 5.25 per 100,000 population, and the prevalence of NETs in the United States exceeds 100,000 individuals [7]. The incidence and prevalence of NETs is steadily increasing attributable, at least in part, to the increasing of awareness, widespread availability of imaging, improved diagnostic tools and a change in definition.

The propensity to metastasize depends on the location of the primary tumor, histologic grade, and tumor size. Most neuroendocrine tumors arising from the small intestine have a propensity to metastasize regardless of the size. More common sites of metastases include the regional lymph nodes, liver, bone, and lung. Median overall survival for localized ileal neuroendocrine tumor is 111 months compared to 56 months in case of metastatic ileal NET [8].

Skeletal metastases from well differentiated neuroendocrine tumors is a rare event and only a few cases have been reported in the literature, although peculiar metastatic site to the orbital muscles is described in small case series [9-12]. The exact risk of muscle metastasis remains unclear and impact on survival is unknown. Given the indolent nature of NETs we can only speculate that skeletal metastases from NETs have a better prognosis, if not the best prognosis compared with skeletal metastases from other solid tumors. In our review of the literature, we found 3 cases of skeletal muscles metastases from well differentiated neuroendocrine tumor (Table 1). In all cases, asymptomatic muscle metastases were detected by somatostatin receptor scintigraphy (OctreoScan). Including our case, the ileocecal tumor was a primary disease site in three patients with metastases involving lower extremity muscle. Two patients had concomitant liver metastases and the other 2 did not have any other site of metastases. The time from initial NET diagnosis to diagnosis of skeletal metastasis was 2.5 months to 5 years. There have been no published survival data for patients with skeletal metastasis from NETs and a lack of clear guidance for the clinical management of such patients.

**Table 1:** Summary of Case Reports

	Case 1 Quan GM, et al [9]	Case 2 Tiktinsky E, et al [10]	Case 3 Caobelli F, et al [11]	Our case
Age/Sex	53/M	43/M	70/M	66/M
1° site	Ileocecal	Lung	Ileal	Ileocecal
Size of 1° tumor	Not reported	Not reported	Not reported	3.4 cm
Soft tissue mets	Popliteus muscle	Transverse abdominal muscle	Thigh muscle	Vastus intermedius
Other site of metastasis	Liver	None	None	Liver
Time between initial diagnosis and presentation	2.5 years	2 months	5 years	6 months
Octreoscan	(+)	(+)	(+)	(+)
Biopsy	Metastatic malignant carcinoid	Atypical carcinoid	Carcinoid	Well differentiated neuroendocrine tumor
Treatment	Not reported	Not reported	Not reported	Octreotide LAR

OctreoScan is highly specific (more than 90% specificity in most studies) for detection of neuroendocrine tumors due to high concentrations of somatostatin receptors, both in functioning and non-functioning tumors [13]. Interestingly enough, despite the high specificity of OctreoScan for neuroendocrine tumors, all the cases that were reported were biopsy confirmed. Radiological diagnosis of skeletal muscles metastasis such as combination of contrast enhancing CT and Octreoscan may be highly specific for NET and may not require tissue diagnosis.

According to the largest NET epidemiological study, improvement in survival duration among patients with metastatic NETs has been noted recently. One possible explanation is the introduction of octreotide in 1987 that improved the control of carcinoid syndrome and changed the natural history of NETs [10]. Due to longer survival times, new metastatic patterns, such as skin, muscle and bone metastases, may become more important features of carcinoid disease.

### Conclusion

In summary, we report a case of metastatic well-differentiated neuroendocrine tumor to the vastus intermedius muscle detected by Octreotide scan. Due to the rarity of skeletal muscles metastases, its prognosis and management is uncertain at this time. OctreoScan is increasingly used for NETs detection, follow-up and monitoring; therefore, more cases of skeletal muscles metastasis are likely to be reported.
